# The Glitazone Class of Drugs as Carbonic Anhydrase Inhibitors—A Spin-Off Discovery from Fragment Screening

**DOI:** 10.3390/molecules26103010

**Published:** 2021-05-18

**Authors:** Sarah L. Mueller, Panagiotis K. Chrysanthopoulos, Maria A. Halili, Caryn Hepburn, Tom Nebl, Claudiu T. Supuran, Alessio Nocentini, Thomas S. Peat, Sally-Ann Poulsen

**Affiliations:** 1 Griffith Institute for Drug Discovery, Griffith University, Nathan, Brisbane, QLD 4111, Australia; sarah.muller2@griffithuni.edu.au (S.L.M.); panos@artisancells.com (P.K.C.); m.greenup@griffith.edu.au (M.A.H.); 2 ARC Centre for Fragment-Based Design, Griffith University, Nathan, Brisbane, QLD 4111, Australia; 3 CSIRO, Biomedical Manufacturing Program, Parkville, Melbourne, VIC 3052, Australia; Tom.Nebl@csiro.au (T.N.); tom.peat@csiro.au (T.S.P.); 4 Waters Australia Pty Ltd., Rydalmere, NSW 2116, Australia; Caryn_Hepburn@waters.com; 5 Dipartimento Neurofarba, Sezione di Scienze Farmaceutiche Nutraceutiche, Università Degli Studi di Firenze, Sesto Fiorentino, 50019 Florence, Italy; claudiu.supuran@unifi.it (C.T.S.); alessio.nocentini@unifi.it (A.N.)

**Keywords:** diabetes, carbonic anhydrase, metalloenzyme, glitazones, fragment-based drug discovery, zinc binding group, native mass spectrometry, protein crystallography

## Abstract

The approved drugs that target carbonic anhydrases (CA, EC 4.2.1.1), a family of zinc metalloenzymes, comprise almost exclusively of primary sulfonamides (R-SO_2_NH_2_) as the zinc binding chemotype. New clinical applications for CA inhibitors, particularly for hard-to-treat cancers, has driven a growing interest in the development of novel CA inhibitors. We recently discovered that the thiazolidinedione heterocycle, where the ring nitrogen carries no substituent, is a new zinc binding group and an alternate CA inhibitor chemotype. This heterocycle is curiously also a substructure of the glitazone class of drugs used in the treatment options for type 2 diabetes. Herein, we investigate and characterise three glitazone drugs (troglitazone **11**, rosiglitazone **12** and pioglitazone **13**) for binding to CA using native mass spectrometry, protein X-ray crystallography and hydrogen–deuterium exchange (HDX) mass spectrometry, followed by CA enzyme inhibition studies. The glitazone drugs all displayed appreciable binding to and inhibition of CA isozymes. Given that thiazolidinediones are not credited as a zinc binding group nor known as CA inhibitors, our findings indicate that CA may be an off-target of these compounds when used clinically. Furthermore, thiazolidinediones may represent a new opportunity for the development of novel CA inhibitors as future drugs.

## 1. Introduction

**Figure 1:**
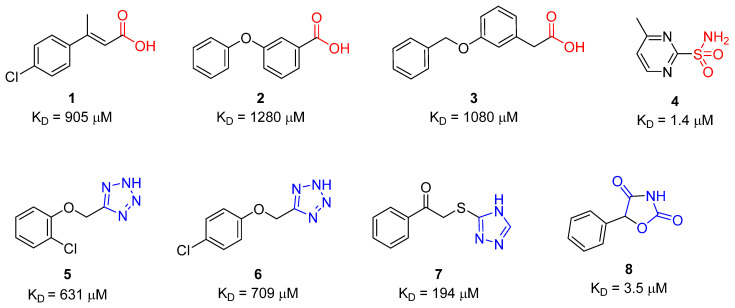
CA II hit fragments identified using surface plasmon resonance and native state mass spectrometry [18]. Red—known zinc binding groups of carboxylic acid (**1**–**3**) and primary sulfonamide (**4**). Blue—Novel zinc binding groups—tetrazole (**5**–**6**), 1,2,4-triazole (**7**) and 3-unsubstituted oxazolidinedione (**8**).

Fragment-based drug discovery (FBDD) continues to be widely applied in academia and the biotechnology and pharmaceutical sectors as an established drug lead-finding technology [1,2,3,4,5,6]. An essential component of the FBDD workflow is the screening of fragment libraries against a protein of interest to identify fragment hits. The screening hits are then validated and triaged for development into lead compounds that may progress through the drug development pipeline. It is estimated that 10% of human proteins utilise zinc to support structure, function, or both [7], therefore, it is not surprising that fragment screening campaigns with zinc metalloproteins have been pursued. For example, Cohen and colleagues have used fragment libraries with a composition bias toward known metal binding pharmacophores for screening with a range of zinc metalloproteins [8,9,10]. Our team has a long-standing interest in discovery of novel inhibitors of carbonic anhydrases (CA, EC 4.2.1.1), a family of zinc metalloenzymes that catalyse the reversible hydration of CO_2_ to give HCO_3_^−^ and H^+^ [11,12,13,14,15,16,17]. To this end, we have previously performed a fragment screening campaign with CA II using an unbiased 720-member fragment library [18,19]. The biophysical techniques applied for primary screening to identify the CA II fragment hits were surface plasmon resonance (SPR) and native state mass spectrometry, followed by protein X-ray crystallography for fragment hit validation and binding pose determination [18,19]. From this campaign, eight hit fragments were identified, including those with groups that have a known zinc binding propensity (carboxylates **1**–**3** and sulfonamide **4**), as well as three previously unknown zinc binding groups: 5-substituted tetrazoles (**5** and **6**), 3-substituted 1,2,4-triazole (**7**) and 3-unsubstituted oxazolidinedione (**8**), Figure 1. Compounds **5**–**8** are heterocycles with an acidic ring nitrogen and X-ray crystallography showed a direct interaction between the N-1 atom of **5** and **6**, and with the ring N of **8** with the CA II active site zinc [18,19]. An X-ray crystal structure was not determined for **7**.

The binding strength of fragment **8** was a standout, exceeding that of all other fragment hits. With a K_D_ = 3.5 µM as measured by SPR, the binding strength of **8** is similar in magnitude to that of the classic primary sulfonamide CA inhibitor, represented by fragment **4** (K_D_ = 1.4 µM). X-ray crystallography showed that the acidic ring nitrogen of **8** bound to the CA II active site zinc, a hydrogen bond between the ring oxygen atom and the active site Thr199 backbone nitrogen, and a second hydrogen bond between the ring nitrogen and the Thr199 side chain hydroxyl (PDB entries 5TXY and 5TY8), Table 1. This binding pose indicates that **8** is an isostere of the classic primary sulfonamide anion when bound within the CA II active site. To demonstrate, the deprotonated sulfonamide of benzene sulfonamide **9** coordinates to the CA II active site zinc cation, also forming two hydrogen bonds with the active site Thr199 (PDB entry 4YX4), Table 1 [20]. Another previously unknown zinc binding chemotype was subsequently discovered in thiazolidinedione **10**, which differs to oxazolidinedione **8** by having the ring oxygen of **8** replaced with a sulfur. The structure of the CA II complex with **10** was determined by protein X-ray crystallography, Table 1. In contrast to oxazolidinedione **8**, there is no hydrogen bond formed between the ring sulfur and the Thr199 backbone nitrogen, a finding that is consistent with the weaker affinity measured for **10** (K_D_ = 32.9 µM) compared to **8** (K_D_ = 3.5 µM).

**Figure 2:**
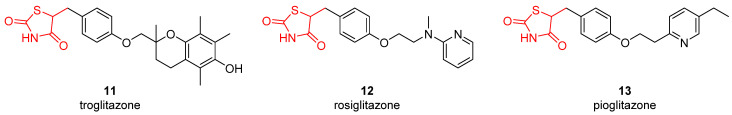
Thiazolidinedione drugs, also known as glitazones, used in management and treatment of type 2 diabetes mellitus. The thiazolidinedione heterocycle is shown in red.

Approved drugs and small molecule inhibitors that target CAs are almost exclusively primary sulfonamides (R-SO_2_NH_2_) or their isosteres, primary sulfamates or sulfamides (R-XSO_2_NH_2_), where X is O or NH, respectively. The deepening understanding of the biology of different CA isozymes in recent years has put a spotlight on the importance of identifying alternative CA inhibitor chemotypes to complement the classic CA drug and inhibitor chemotypes. We recognised the thiazolidinedione, where the ring nitrogen carries no substituent, is a substructure of the glitazone class of drugs, one of the treatment options for type 2 diabetes [21]. Given that thiazolidinediones are not credited as a zinc binding group, or known as CA inhibitors, we undertook this study to further characterise these intriguing features. Herein we report our approach and findings with the evaluation of three glitazone drugs: troglitazone (**11**), rosiglitazone (**12**) and pioglitazone (**13**), Figure 2.

Thiazolidinediones are insulin-sensitising compounds that have been used for management and treatment of type 2 diabetes mellitus [22]. This group of synthetic compounds were found to be high affinity ligands for the peroxisome proliferator-activated receptor γ (PPARγ) [23], a family of ligand-activated transcription factors of nuclear hormone receptors that are involved in the regulation of energy homeostasis. PPARγ usually resides in the cell cytoplasm and is most abundant in adipose tissue, but is also expressed in the kidney, stomach, liver, brain and macrophages [24,25,26]. Upon activation by ligands such as thiazolidinediones, cytosolic PPARγ translocates to the nucleus and binds to specific DNA segments to regulate gene expression linked to glucose homeostasis, fatty acid storage, adipocyte differentiation and anti-inflammatory pathways [27,28]. Although effective in relation to modulating insulin sensitivity, glitazones have been associated with a range of severe side effects, including heart failure, idiosyncratic hepatotoxicity, fluid retention and weight gain [21,29,30]. They have been carefully scrutinised by the US Food and Drug Administration and other regulatory authorities for medicines since their implementation [21,29,30]. Troglitazone **11**, the first of the glitazone drug family approved for use in 1997 as treatment for type 2 diabetes mellitus. It was withdrawn from the market in the United Kingdom the same year, and in the United States within 3 years due to reports of severe liver damage and deaths from acute liver failure [30,31,32]. Rosiglitazone **12** and pioglitazone **13** were approved as either monotherapies or for use in combination with metformin or sulfonylureas to manage type 2 diabetes mellitus [30,33]. Rosiglitazone **12** is similarly associated with numerous adverse side effects, including heart failure, and was withdrawn from the market in Europe in 2010 [34]. Pioglitazone **13** has been linked to an increased risk of bladder cancer and has therefore been either relabelled or withdrawn from the market, depending on the country of use [35,36]. There are reported off-label uses of the glitazone thiazolidinediones, e.g., in the treatment of polycystic ovary syndrome to improve endothelial function and ovulation and in the treatment of chronic granulomatous disease [37,38]. A structurally similar chemical family of compounds, rhodanines, contain a thiocarbonyl in place of the carbonyl group of thiazolidinediones. Rhodanines are considered pan assay interference compounds (PAINS) and are recognised by the PAINS chemical structure and physicochemical property filter as a problematic chemotype for drug discovery assays [39]. It is important to highlight that rhodanines differ to thiazolidinediones, as the structural difference is sufficient for the glitazone chemical structures to pass through the PAINS filter without any alerts [40].

## 2. Results and Discussion

### 2.1. Native Mass Spectrometry

To establish if the glitazone drugs **11**–**13** bind to the human CA II enzyme (hCA II), we employed native state nanoelectrospray ionisation mass spectrometry (nanoESI-MS) with test samples comprising hCA II (10 μM) and drug (10 μM). The noncovalent hCA II drug complexes were observed for each of **11**–**13** together with some unbound hCA II protein. The noncovalent complexes are at higher *m/z* values than the unbound protein; the difference between the *m/z* values of the complex and the unbound protein is consistent with the molecular weight of the bound drug. To quantify the binding, we determined the ratio of the (hCA II + drug) peaks relative to total protein peaks (bound to drug (hCA II + drug) and unbound (hCA II unbound)) and expressed this as a percentage [19]. The calculated value for troglitazone **11** is 86.4%, rosiglitazone **12** is 75.1% and pioglitazone **13** is 24.2%. Overall, these findings provide confirmation that the glitazone drug class bind to hCA II. Notably, **11** and **12** have similar binding that is approximately threefold stronger than the binding observed with **13**.

### 2.2. Protein X-ray Crystallography

**Figure 3:**
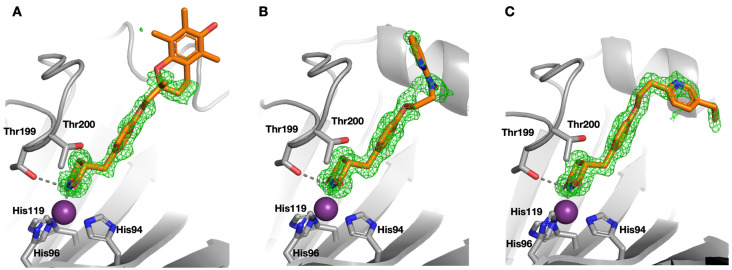
Crystal structure of troglitazone **11** (**A**), rosiglitazone **12** (**B**) and pioglitazone **13** (**C**) with hCA II. The Zn(II) ion (purple sphere) with the three coordinating histidines His94, His96 and His 119 are shown. The glitazone drugs are bound to the zinc ion (2.0 Å) supported by hydrogen bond formation to Thr199 (3.1 Å). Difference density maps are represented at a 3 σ contour level.

The thiazolidinedione drugs **11**–**13** comprise a hydrophilic head group (the thiazolidinedione heterocycle) and a lipophilic tail group separated by an aliphatic linker. The compounds are used clinically as mixtures of isomers. Specifically, the asymmetric C-5 carbon of the drugs is prone to spontaneous racemisation in solution, while **11** contains a second chiral centre and thus comprises four isomers (*RR*, *RS*, *SR*, *SS*). To investigate the binding pose with hCA II, we determined the protein X-ray crystal structure of hCA II in complex with each of the drugs. They all bound to hCA II via an interaction of their acidic imide nitrogen with the active site zinc, Figure 3. This is similar to that described for the imide nitrogen of the lead thiazolidinedione fragment **10** [19]. While a hydrogen bond is also formed between the Thr199 side chain hydroxyl and the imide nitrogen (3.1 Å), most of the interaction takes place between the nitrogen and the zinc ion (2.0 Å), Figure 3.

In each drug-hCA II complex X-ray crystal structure there is clear electron density for the compound close to the zinc ion active site after the first round of refinement. The difference density map shows density for the thiazolidinedione ring and the middle benzene ring, but little to no density beyond the aliphatic linker, Figure 3. Protein X-ray crystallography data collection and structure refinement statistics are in Table S1. Since the data are complete and high resolution, we assume that this part of the compounds is present in several different conformations and is therefore not resolved. Furthermore, as troglitazone **11** contains a second chiral centre beyond the aliphatic linker, with little density in the crystal structure, we cannot say with certainty whether *R,S* or *R,R* troglitazone is bound in the protein.

**Figure 4:**
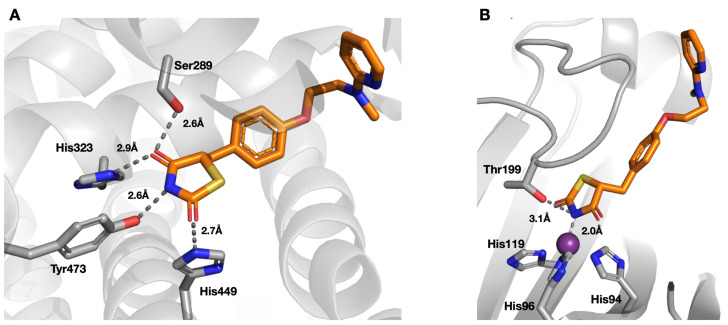
Comparison of binding interactions of rosiglitazone **12** with PPARγ (**A**) and with hCA II (**B**). In complex with PPARγ, the thiazolidinedione moiety of **12** is bound via hydrogen bonds formed between His323, His449, Tyr473 and Ser289. In comparison with hCA II, there is a close ionic bond between the imide nitrogen and the active site zinc cation and only one hydrogen bond formed between the imide nitrogen and Thr199.

These findings have both similarities and differences to the binding interactions reported for the structure of PPARγ in complex with these thiazolidinediones. Since PPARγ is not a metalloprotein, drug binding occurs largely via hydrogen bonds and van der Waals interactions in the PPARγ binding site [41]. The crystal structure of PPARγ bound to rosiglitazone **12** (PDB ID 4EMA) [42] shows hydrogen bond formation between the imide nitrogen and the Tyr473 hydroxyl group (2.6 Å). Another two hydrogen bonds are formed between His323 and His449 and the two carbonyls of **12** at 2.7 Å and 2.9 Å, respectively, Figure 4. Ser289 forms an additional hydrogen bond to one of the carbonyl groups, but also shows steric interactions with the ligand that probably counteract the hydrogen bonding effect, Figure 4. Otherwise, there are no hydrophilic interactions with the rest of the ligand, the major binding interactions taking place with the thiazolidinedione heterocycle component, which is similar to what is found with these thiazolidinedione-based drugs when bound to hCA II (see above).

### 2.3. Hydrogen–Deuterium Exchange Mass Spectrometry

To investigate the conformational dynamics of the backbone of hCA II between the bound and unbound state, we examined hCA II by hydrogen–deuterium exchange mass spectrometry (HDX-MS). HDX-MS measures the exchange rate of the amide hydrogen atoms of the protein backbone, which depends on the accessibility of the solvent and hydrogen bonding, providing valuable information about protein dynamics and conformational changes. We compared the HDX behaviour of hCA II in the presence and absence of troglitazone **11**, rosiglitazone **12** and pioglitazone **13** (93.5% hCA II sequence coverage, Figure S1) Deuterium uptake is decreased in two regions (residues 49–66 and 197–204, *p*-value < 0.01) upon ligand binding, suggesting induced protein stability with the ligandprotein interaction. A difference is noticeable after 600 and 6000 s, with consistent results for troglitazone **11** and rosiglitazone **12** (heat map, Figures S2 and S3). In the case of pioglitazone **13**, the results differ slightly, but overall show the same tendency (heat map, Figure S4). The different results could be due to the higher insolubility of **13** compared to **11** and **12,** and the additional lower inhibitory effect (*vide infra*) of *K*_i_ of 7.1 μM for **13** versus *K*_i_ values of 1.3 and 0.75 µM for **11** and **12**, respectively, Table 2.

**Figure 5:**
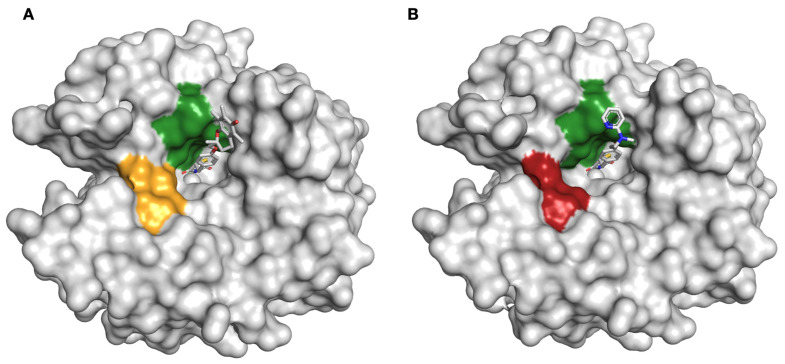
(**A**). Crystal structure of hCA II bound to troglitazone **11** (shown in grey surface) coordinated to the zinc ion (shown as a grey sphere). Significant regions are coloured in green (deuterium uptake after 6000 s) or orange (deuterium uptake after 600 s). (**B**). Crystal structure of hCA II bound to rosiglitazone **12** (shown in grey surface) coordinated to the zinc ion (shown as a grey sphere). Significant peptides are coloured in green (deuterium uptake after 6000 s) or red (deuterium uptake after 30 s).

With troglitazone **11** there is a difference in deuterium uptake in peptides 197–203, 198–203 and 198–204 after 6000 s and in peptide 58–66 after 600 s (Figure S5A/B). These findings are comparable with the compound–protein interactions visualised in the crystal structure for **11**, Figure 5A. Due to the way the compound is bound to the zinc ion active side, and by formation of a hydrogen bond with Thr199, the glitazone drugs are in close contact with peptide region 197–204, which makes this region less accessible for solvent molecules. By comparison, peptide 58–66 shows a relatively small but significant change in deuterium uptake. While this sequence is further away from the active site, it is known to play an important role in the catalytic reaction of the enzyme. Specifically, this region contains the residue His64, also known as the proton shuttle residue, which interacts with the zinc-bound solvent via a hydrogen bonding network to promote efficient proton transfer. During catalysis, this region undergoes different conformational changes, which could affect solvent exchange. Similar results are observed for **12** as visualised in the crystal structure in Figure 5B, with peptides 197–203, 197–204, 198–203, 198–204 and 49–62 showing slower deuterium uptake than unbound hCA II, with comparable rationale to **11** (Figure S6A/B).

### 2.4. Carbonic Anhydrase Inhibition

**Figure 6:**
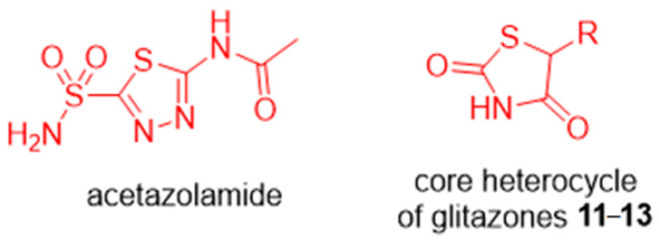
Comparison of the clinically used CA inhibitor acetazolamide with the heterocyclic core of glitazone drugs **11**–**13**.

CA enzyme inhibition data was determined by measuring the CA-catalysed hydration of CO_2_ in the presence of **11**–**13**. In addition to the ubiquitous hCA II, all other catalytically active hCA enzymes (I, IV, VA, VB, VI, VII, IX, XII, XIII and XIV) were assessed, providing a comprehensive profile of CA enzyme inhibition, Table 2. The CAs share similar active site structure and architecture, comprising a conical cavity that is 15 Å wide and 15 Å deep, with the catalytic zinc ion located at the bottom of this cavity and coordinated by three histidine residues. Different CAs have different expression profiles and cell locations [11]. All glitazones are weaker CA inhibitors than acetazolamide, a clinically used CA inhibitor included for comparison, Figure 6, Table 2. For hCA II, the inhibition constant values determined are consistent with the binding strength trend determined by mass spectrometry, with **11** and **12** exhibiting similar potency (*K_i_* values of 1.3 and 0.75 μM, respectively) while **13** was approximately sevenfold lower in potency (*K_i_* = 7.1 μM). Similar to hCA II, but potentially more significant for future drug discovery, the glitazones are most potent in their inhibition of hCA IX and XII (*K_i_* values ranging from 0.75–3.7 μM), the two extracellular facing membrane-bound CAs which are induced by hypoxia and are overexpressed in many solid and hypoxic tumors where they contribute pH regulation of the cancer microenvironment, supporting invasion, metastasis, and drug resistance [43,44,45,46]. The next most potent inhibition values are for hCA VII (*K_i_* values ranging from of 2.5–6.1 μM, acetazolamide *K_i_* = 0.003 μM). hCA VII is one of the least investigated Cas; notably, it is not ubiquitously expressed with localisation, mainly in brain tissues of mammals [47]. A comparison between hCA VII and hCA II has identified 7 residue differences (S65A, Q67N, D69E, K91I, A135V, S136Q, S204L) among the 23 residues within the active site cavity [47]. It has been suggested that this high sequence and structural similarity is likely responsible for the minimal differences found in the catalytic activity of these two isozymes [47]. Here, we propose that this further extends to the minimal differences in observed active site compound binding strength between these isozymes. The three glitazone drugs were low micromolar inhibitors of hCA VA (*K_i_* values ranging from 5.3–12.4 μM), and weaker micromolar inhibitors of hCA VB (*K_i_* value range of 28.9–40.9 μM). hCA VA and VB are located in the mitochondria of some cell types and are involved in energy metabolism [48]. Furthermore, they are considered a validated target for antiobesity drug development, in which they have been proposed to have an involvement in insulin secretion, however this mechanism is not yet fully understood [48]. The compounds also have a similar inhibition profile of hCA VI, XIII and XIV, with compound **12** being the most potent for these three isozymes (*K_i_* values of 2.3–7.4 μM, acetazolamide *K_i_* values of 0.011–0.041 μM). Finally, the compounds are very weak inhibitors of the transmembrane hCA IV (*K_i_* values of 58.9 to >100 μM, acetazolamide *K_i_* = 0.074 μM). hCA IV is known for widespread roles, including epilepsy, renal disorders and eye disorders [49]. The appreciable inhibition of the glitazones for the different CA isozymes highlights the potential for CA-mediated off-target effects of these drugs when used clinically, and given the reported polypharmacology observed for these compounds, this may provide an avenue for further understanding of their mechanism of action.

Surface plasmon resonance (SPR) is a technique employed for the study of ligand binding interactions with proteins, providing a measurement of quantitative binding affinities. SPR binding data with PPARγ, considered the therapeutic target of glitazones, have previously been reported for troglitazone **11** (K_D_ 0.274 ± 0.014 µM) [50] and rosiglitazone **12** (K_D_ 0.321 ± 0.015 µM) [51]. While not directly comparable to CA enzyme inhibition, it is interesting to note that the SPR K_D_ values for **11** and **12** are similar in scale, both being just below 1 µM, and not substantially lower concentration than those required to inhibit hCA II (*K_i_* values 1.3 and 0.75 μM, respectively).

## 3. Materials and Methods

The three glitazone compounds were purchased from Merck (Darmstadt, Germany) as solids >98% purity: (±)-5-[4-[(6-Hydroxy-2,5,7,8-tetramethylchroman-2-yl)methoxy]benzyl]-2,4-thiazolidinedione (troglitazone, **11**); 5-[[4-[2-(methyl-2-pyridinylamino)ethoxy]phenyl]methyl]-2,4-thiazolidinedione (rosiglitazone, **12**) and 5-[[4-[2-(5-ethyl-2-pyridinyl)ethoxy]phenyl]methyl]-2,4-thiazolidinedione monohydrochloride (pioglitazone hydrochloride, hydrochloride salt of **13**).

### 3.1. Native Mass Spectrometry

Glitazone compound dry stocks were dissolved in DMSO to give 5 mM stock solutions. hCA II was concentrated and buffer exchanged into 10 mM NH_4_OAc pH 7.0 using Amicon Ultra 0.5 centrifugal filters (Merck Millipore, Sydney, NSW, Australia) and immediately prior to mass spectrometric analysis, described by us previously [19]. Protein and test compound solutions were mixed to provide a protein/ligand ratio of 1:1 (10 µM) with a 0.5% DMSO final concentration. Samples were incubated for 10 min then infused using a Triversa Nanomate (Advion BioSciences, Ithaca, NY, USA) automated nanoESI interface coupled with a Bruker solariX XR^TM^ 12.0 Tesla Fourier Transform Ion Cyclotron Resonance Mass Spectrometer (FT-ICR MS) fitted with a ParaCell^TM^ (Bruker Daltonics Inc., Billerica, MA). Spraying conditions were optimised for the best mass spectrometric signal intention, signal to noise ratio and spray duration [18,19]. The instrument was calibrated using perfluorohexanoic acid (PFHA) and pure hCA II. Data were acquired for 30 scans, over *m/z* 500–10,000 with the quadrupole set at *m/z* 600, Skimmer 1 voltage of 30 V, drying gas temperature of 100 °C, nebulizer gas at 2 bar, capillary voltage of 3500 V, spray shield voltage of 500 V, collision voltage (entrance) of −3.0 V, ion accumulation time of 0.001 s and flight time of 2.1 msec. Mass spectra were processed with Bruker Compass DataAnalysis 4.2 (Bruker Daltonics Inc., Billerica, MA). The mass spectrometry fragment binding (FB_MS_) percentage was calculated as the ratio of the measured intensity of the fragment-bound protein (PL) peak to the sum of the bare protein (P) and PL peaks for each spectrum, as previously reported by us [19].

### 3.2. Protein X-ray Crystallography

The human CA II enzyme was expressed and purified as previously described [15]. The protein was concentrated to 4 mg/mL and set in MRC-2 (Molecular Dimensions, Sheffield, United Kingdom) crystallisation 96-well plates with a Phoenix crystallisation robot (Art Robbins Instruments, Sunnyvale, CA, USA) and a protein/reservoir ratio of 1:1 (250 nL/250 nL). The plate was incubated at 20 °C and the reservoir condition consisted of 1.5 M tripotassium citrate with 0.1 M Tris buffer at pH 8.3. The compound was dissolved in 2-pyrrolidinone and added to the crystallisation drop after crystals had formed, incubated overnight, and harvested for data collection. A total of 360° were taken at the MX-1 beamline of the Australian Synchrotron [52]. The data were indexed using XDS [53] and scaled using Aimless [54]. Molecular replacement was performed using Phaser [55] and PDB ID 6ODZ as the initial starting model. The model was manually rebuilt using Coot [56] and refined using REFMAC5 [57]. The compound dictionary file defined by elBOW [58] was placed in density using the program LigandFit [59] (both from the Phenix software package) and further refined using REFMAC5 [57].

### 3.3. Hydrogen–Deuterium Exchange Mass Spectrometry

HDX labelling of hCA II with and without inhibitor was performed at 20 °C for periods of 0, 10, 30, 60, 600 and 6000 s using a LEAP HDX-2 Automation manager. Purified protein (30 µM) was incubated with an inhibitor (10–100 µM) for 24 h before the hydrogen–deuterium exchange reaction. Subsequently, 3 µL of the protein sample was transferred to 57 µL of deuterated PBS buffer and incubated for the respective time. Quenching was performed by adding 50 µL of the deuterated protein to 50 µL of 50 mM PBS pH 2.5 at 0 °C. For online pepsin digestion, 80 µL of the quenched sample was loaded onto a chilled ACQUITY UPLC M-Class HDX Manager (Waters, Milford, MA, USA) and passed over an immobilised 2.1 × 30 mm Enzymate BEH pepsin column (Waters, Milford, MA, USA) equilibrated in 0.1% formic acid in water at 100 µL/min. Proteolysed peptides were captured and desalted by a C18 trap column (VanGuard BEH; 1.7 μm; 2.1 × 5 mm; Waters, Milford, MA, USA) and eluted with acetonitrile and 0.1% formic acid gradient (5% to 40% 7 min, 40% to 95% 1 min, 95% 2 min) at a flow rate of 40 μL/min using an ACQUITY UPLC BEH C18 analytical column (1.7 μm, 1 × 100 mm, Waters, Milford, MA, USA) delivered by ACQUITY UPLC M-Class Binary Solvent Manager (Waters, Milford, MA, USA).

To ionise peptides, an electrospray ionisation source sprayed onto SYNAPT G2-Si mass spectrometer (Waters) was used. Data were acquired in MS^E^ mode and peptides from non-deuterated samples were identified using Protein Lynx Global Server (PLGS) v3.0 (Waters, Milford, MA, USA). Selected peptides were further screened, and deuterium uptake values were calculated for each peptide using DynamX 3.0 (Waters, Milford, MA, USA). Deuterium exchange experiments were performed in triplicate for each of the timepoints. HDX-MS data were analysed using the HD-eXplosion software tool for the visualization of hydrogen–deuterium exchange data with statistical filtering [60].

### 3.4. Carbonic Anhydrase Inhibition

An Applied Photophysics stopped-flow instrument was used for assaying the inhibition of the CA catalysed CO_2_ hydration reaction [61]. Phenol red (0.2 mM) was used as indicator, monitored at the absorbance maximum of 557 nm. Reactions were carried out in a HEPES (10 mM, pH 7.4) buffer and NaClO_4_ (10 mM), for maintaining the ionic strength constant, the ClO_4_^-^ anion is not inhibitory against most CA isoforms at 25 °C [62]. The reaction was monitored for a period of 10–100 s. The CO_2_ concentrations ranged from 1.7 mM to 17 mM for the determination of kinetic parameters. Inhibitors were tested in the concentration range of 0.01 nM–100 μM, and at least six traces of the initial 5–10% of the reaction have been used for determining the initial velocity. The uncatalyzed rates were determined in the same manner and subtracted from the total observed rates. Stock solutions of inhibitor (1 mM) were prepared in distilled, deionized water with 10–20% (*v*/*v*) DMSO (which is not inhibitory at these concentrations), and dilutions were performed thereafter with the assay buffer. Inhibitor and enzyme solutions were preincubated together for 15 min at room temperature prior to assay to allow for the formation of the enzyme inhibitor complex. The inhibition constants were obtained by nonlinear least squares methods using PRISM 3 (GraphPad Software Inc, San Diego, CA, USA). The curve-fitting algorithm allowed us to obtain the IC_50_ values, working at the lowest concentration of substrate of 1.7 mM), from which *K*i values were calculated by using the Cheng-Prusoff equation. The catalytic activity (in the absence of inhibitors) of the different enzymes was calculated from Lineweaver–Burk plots and represent the mean from at least three different determinations. Enzyme concentrations in the assay system were in the range of 3.8–9.5 nM. Enzymes used here were recombinant ones, prepared and purified as described earlier [63,64,65]. The known CA inhibitor, acetazolamide, was employed as control.

## 4. Conclusions

The thiazolidinedione heterocycle, a substructure found within glitazone drugs, was shown to bind to and inhibit CA activity. Glitazone compounds **11–13** were assessed for binding to CA II using biophysical methods of native mass spectrometry, protein X-ray crystallography and hydrogen–deuterium exchange (HDX) mass spectrometry. The glitazones all displayed appreciable binding and inhibition of different CA isozymes. Given that thiazolidinediones are not previously known as CA inhibitors, our findings indicate that CA may be an off-target of these compounds when used clinically. This relationship has not been investigated; however this will be an important step to assess if CA inhibition may explain some of the side effects observed when patients are treated with glitazone drugs. Furthermore, thiazolidinediones may represent an exciting opportunity for the development of novel CA inhibitors as future drugs.

## Figures and Tables

**Table 1:**
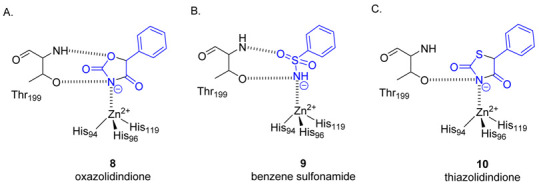
Binding affinity and interactions with CA II active site zinc and amino acid residues observed by protein X-ray crystallography. (**A**). The oxazolidinedione fragment hit, compound **8**. (**B**). The classic primary sulfonamide CA II chemotype **9** [20]. (**C**). The thiazolidinedione fragment hit, compound **10** [19].

Compound	SPR K_D_ (μM)	PDB ID	Protein-Fragment Interaction Distances (Å)		
			Zn-NH	T199OH-NH	T199NH-O1/S1
8	3.5	5TXY and 5TY8	1.9	3.2	3.0
9 [20]	1.2	4YX4	1.9	2.9	3.0
10	32.9	5TYA	2.0	3.1	3.7

**Table 2:** Inhibition data of human CA isoforms CA I, II, IV, VII, IX and XII with troglitazone **11**, rosiglitazone **12**, pioglitazone **13** and the standard sulfonamide inhibitor acetazolamide.

Compound	K_i_(μM) ^a^										
	hCA										
	I	II	IV	VA	VB	VI	VII	IX	XII	XIII	XIV
**11**	57.2	1.3	>100	12.4	28.9	46.7	2.5	1.3	0.92	32.3	16.8
**12**	16.3	0.75	58.9	8.2	40.9	5.9	6.1	2.5	3.7	7.4	2.3
**13**	39.6	7.1	92.1	5.3	36.2	19.8	4.9	0.75	0.85	21.5	6.6
acetazolamide	0.25	0.012	0.074	0.063	0.054	0.011	0.003	0.025	0.006	0.017	0.041

^a^ Mean from three different assays, by a stopped flow CO_2_ hydrase assay (errors were in the range of ±10% of the reported values).

## Data Availability

The structures and structure factors were deposited in the Protein Data Bank (PDB) with accession codes 7M23 (CAII-**11** complex), 7M24 (CAII-**12** complex) and 7M26 (CAII-**13** complex).

## Supplementary Materials

Supplementary Materials for Hydrogen–Deuterium Exchange Mass Spectrometry (Figures S1–S6) and Protein X-ray Crystallography (Table S1) are provided.
