# Supplementary material for: The Glitazone Class of Drugs as Carbonic Anhydrase Inhibitors—A Spin-Off Discovery from Fragment Screening
Source: Molecules. 2021 May 18;26(10):3010. doi: 10.3390/molecules26103010 (PMC8158703; doi:10.3390/molecules26103010)
Supplement: Supplementary file 1 [file molecules-26-03010-s001.zip › molecules-1203853-supplementary.pdf]

molecules

Supplementary Materials

The Glitazone Class of Drugs as Carbonic Anhydrase Inhibitors—A Spin-Off Discovery from Fragment Screening

Sarah L. Mueller [1,2,3], Panagiotis K. Chrysanthopoulos [1], Maria A. Halili [1,2], Caryn Hepburn [4], Tom Nebl [3], Claudiu T. Supuran [5], Alessio Nocentini [5], Thomas S. Peat [3] and Sally-Ann Poulsen [1,2,*]

Contents:

Hydrogen-Deuterium Exchange Mass Spectrometry: Figure S1-6

Protein X-ray Crystallography: Table S1

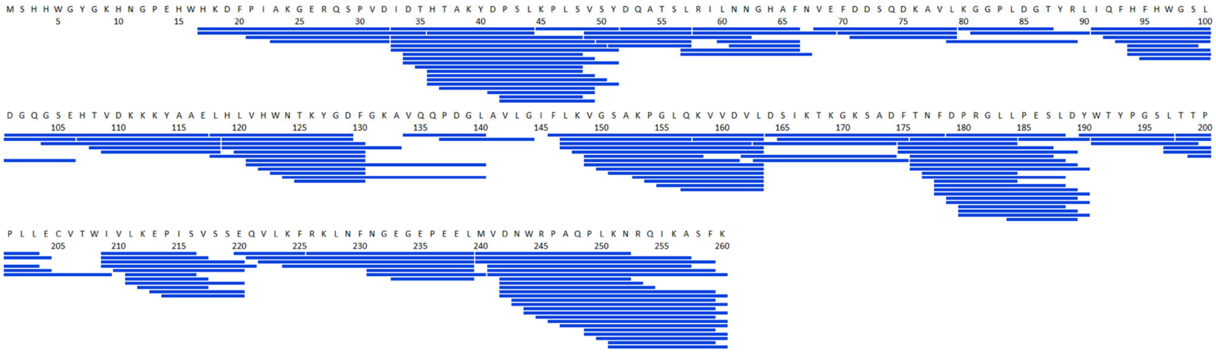

Figure S1. Sequence coverage (93.5%) and coverage redundancy (7.67) of the pepsin digested hCAII. A total of 159 peptides are depicted in blue bars.

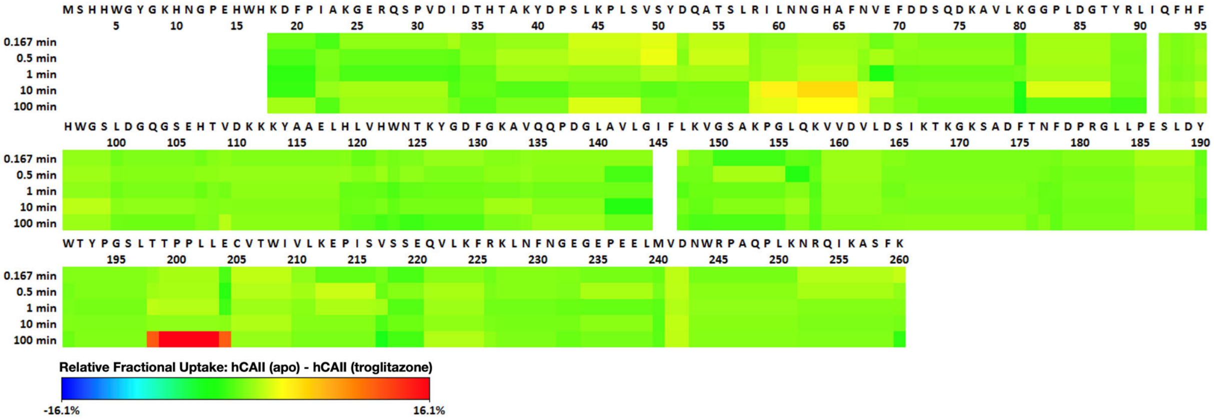

Figure S2. Heat map showing the relative fractional uptake in % apo (no compound) and bound to troglitazone **11**.

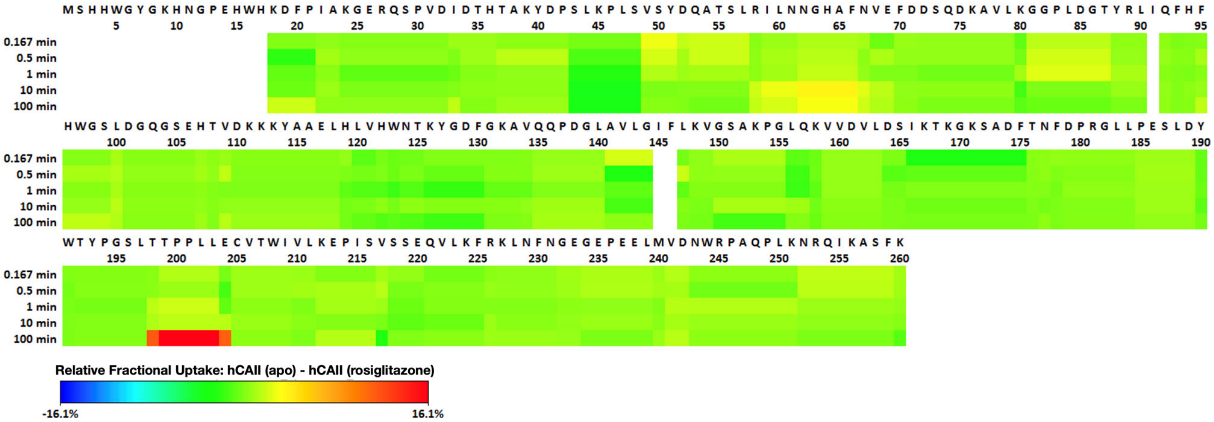

Figure S3. Heat map showing the relative fractional uptake in % between apo (no compound) and bound to rosiglitazone **12**.

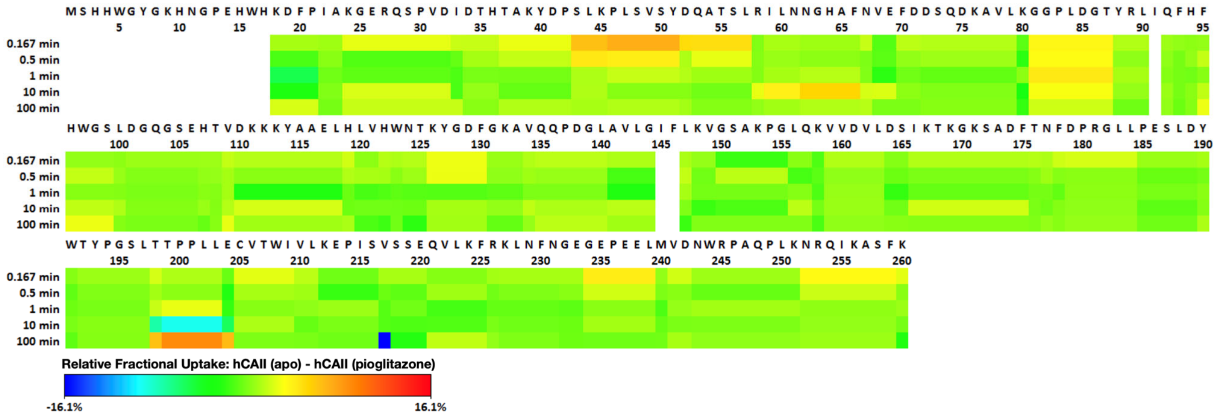

Figure S4. Heat map showing the relative fractional uptake in % between apo (no compound) and bound to pioglitazone **13**.

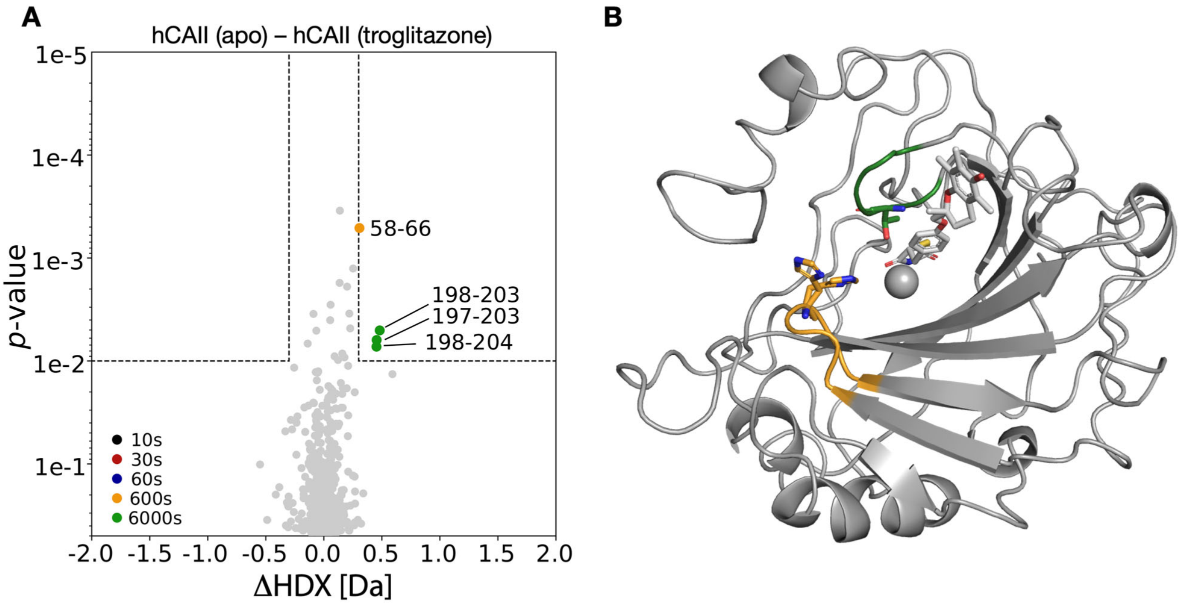

Figure S5. A. Volcano plot [59] showing the average ΔHDX calculated by subtracting either hCA II with troglitazone **11** from apo (protein alone) after 10 s, 30 s, 60 s, 600 s and 6000 s exposure. Significance cut-off values are *p*-value < 0.01 and an average ΔHDX > 0.3 Da. Significant peptides are shown in green (6000 s) or orange (600 s). **B**. Crystal structure of hCA II bound to troglitazone **11** (shown in grey sticks) coordinated to the zinc ion (shown as a grey sphere). Significant regions are coloured in green or orange with proton shuttle His64 (orange) and Thr199 (green) marked as sticks.

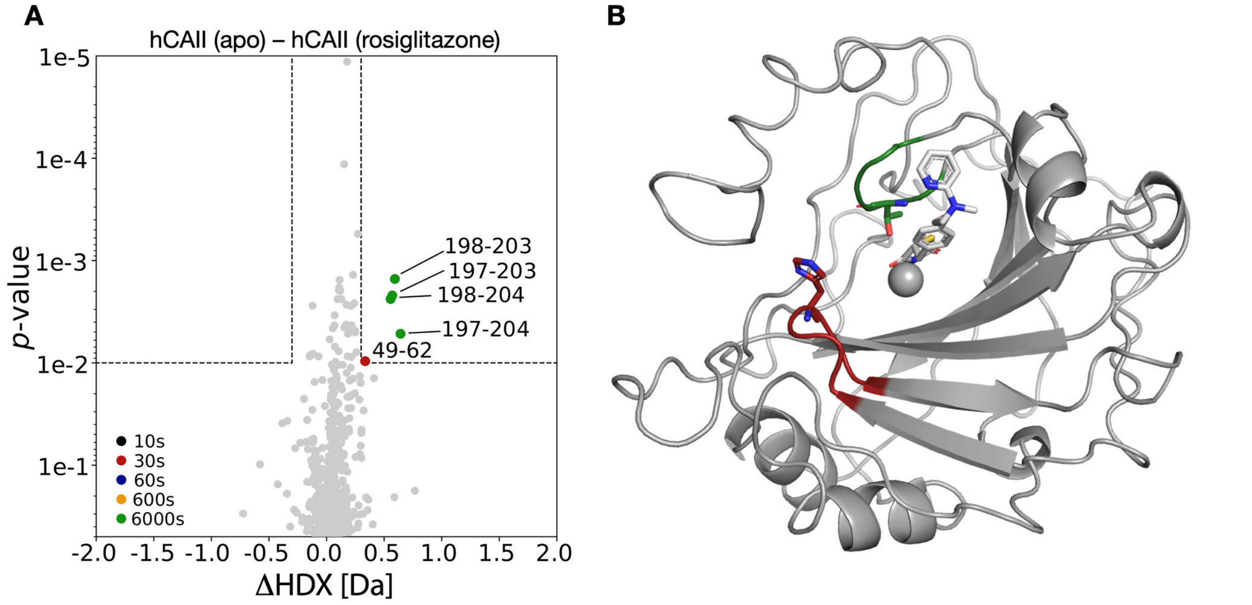

Figure S6. A. Volcano plot [59] showing the average ΔHDX calculated by subtracting either hCA II with rosiglitazone **12** from apo (protein alone) after 10 s, 30 s, 60 s, 600 s and 6000 s exposure. Significance cut-off values are *p*-value < 0.01 and an average ΔHDX > 0.3 Da. Significant peptides are shown in green (6000 s) or red (30 s). **B**. Crystal structure of hCA II bound to rosiglitazone **12** (shown in grey sticks) coordinated to the zinc ion (shown as grey sphere). Significant peptides are coloured in green or red with proton shuttle His64 (red) and Thr199 (green) marked as sticks.

Table S1. Protein X-ray crystallography data collection and structure refinement statistics.

PDB	7M23	7M24	7M26
**Compound**	**11**	**12**	**13**
Space group	$P2_1$	$P2_1$	$P2_1$
Cell dimensions			
a, b, c	42.3, 41.3, 71.7	42.6, 41.3, 71.9	42.4, 41.4, 72.1
alpha, beta, gamma	90, 104.4, 90	90, 104.4, 90	90, 104.4, 90
Resolution (Å)	41.3–1.30	41.3–1.30	41.4–1.30
Resolution-high (Å)	1.32–1.30	1.32–1.30	1.32–1.30
Rmerge	0.071 (0.627)	0.064 (0.443)	0.062 (0.479)
Rpim	0.045 (0.416)	0.040 (0.294)	0.039 (0.320)
CC ½	0.998 (0.825)	0.999 (0.865)	0.999 (0.862)
I/sigI	12.2 (2.8)	14.6 (3.7)	14.8 (3.4)
Completeness (%)	99.1 (97.9)	99.7 (99.3)	99.9 (99.8)
Redundancy	6.6 (6.2)	6.6 (6.2)	6.7 (6.2)
**Refinement**			
Resolution (Å)	39.9–1.30	40.2–1.30	35.7–1.30
Unique reflections	55829	56577	56803
Rwork/Rfree (%)	12.1/15.0	11.6/14.6	11.9/14.5
# atoms	2584	2606	2591
Protein	2273	2268	2276
Metal (Zn)	1	1	1
Ligand	30	25	24
Water	280	313	290
B-factors (Å^2)	14.1	12.8	13.9
Protein	13.8	12.4	13.6
Metal (Zn)	7.3	6.3	6.9
Ligand	36.0	19.7	18.6
Water	27.4	26.5	27.7
r.m.s. deviations			
Bond length (Å)	0.011	0.010	0.011
Bond angle (°)	1.870	1.623	1.708
